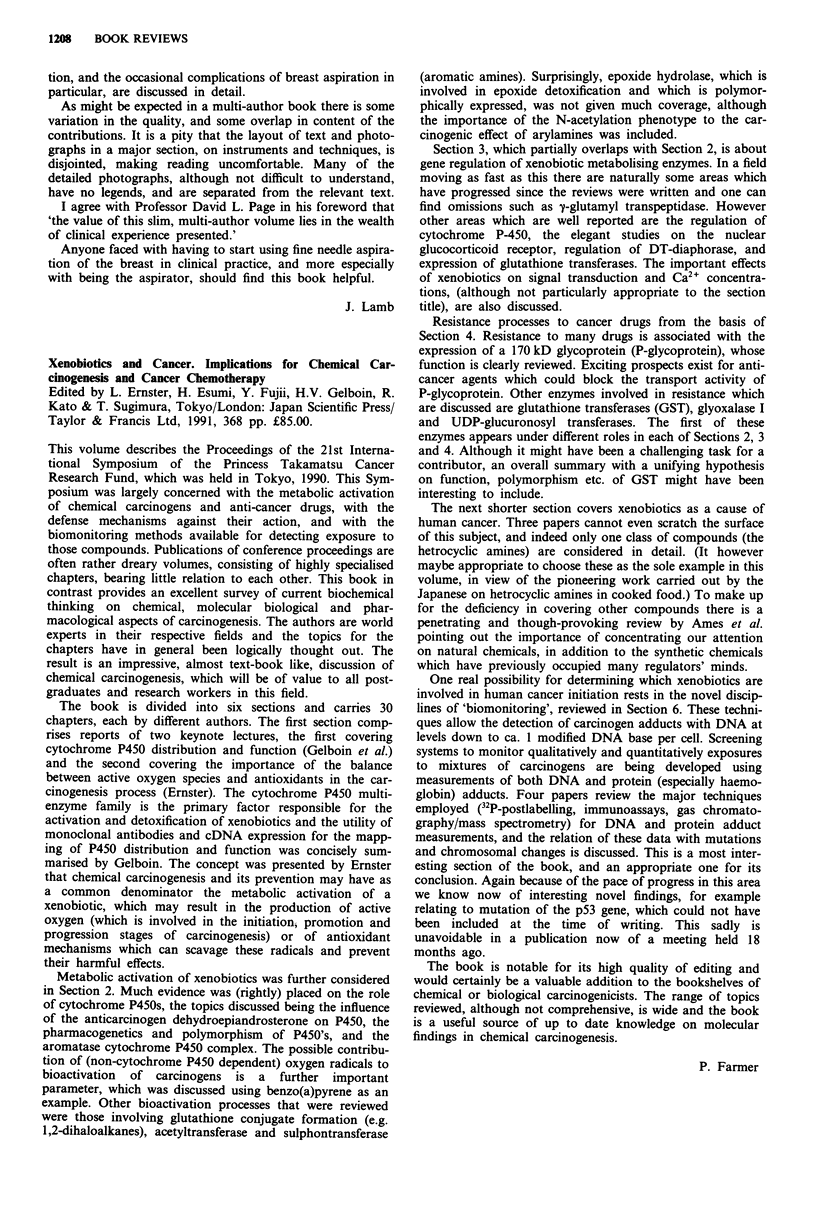# Xenobiotics and Cancer. Implications for Chemical Carcinogenesis and Cancer Chemotherapy

**Published:** 1992-12

**Authors:** P. Farmer


**Xenobiotics and Cancer. Implications for Chemical Carcinogenesis and Cancer Chemotherapy**
Edited by L. Ernster, H. Esumi, Y. Fujii, H.V. Gelboin, R. Kato & T. Sugimura, Tokyo/London: Japan Scientific Press/ Taylor & Francis Ltd, 1991, 368 pp. £85.00.

This volume describes the Proceedings of the 21st International Symposium of the Princess Takamatsu Cancer Research Fund, which was held in Tokyo, 1990. This Symposium was largely concerned with the metabolic activation of chemical carcinogens and anti-cancer drugs, with the defense mechanisms against their action, and with the biomonitoring methods available for detecting exposure to those compounds. Publications of conference proceedings are often rather dreary volumes, consisting of highly specialised chapters, bearing little relation to each other. This book in contrast provides an excellent survey of current biochemical thinking on chemical, molecular biological and pharmacological aspects of carcinogenesis. The authors are world experts in their respective fields and the topics for the chapters have in general been logically thought out. The result is an impressive, almost text-book like, discussion of chemical carcinogenesis, which will be of value to all postgraduates and research workers in this field.

The book is divided into six sections and carries 30 chapters, each by different authors. The first section comprises reports of two keynote lectures, the first covering cytochrome P450 distribution and function (Gelboin *et al.*) and the second covering the importance of the balance between active oxygen species and antioxidants in the carcinogenesis process (Ernster). The cytochrome P450 multienzyme family is the primary factor responsible for the activation and detoxification of xenobiotics and the utility of monoclonal antibodies and cDNA expression for the mapping of P450 distribution and function was concisely summarised by Gelboin. The concept was presented by Ernster that chemical carcinogenesis and its prevention may have as a common denominator the metabolic activation of a xenobiotic, which may result in the production of active oxygen (which is involved in the initiation, promotion and progression stages of carcinogenesis) or of antioxidant mechanisms which can scavage these radicals and prevent their harmful effects.

Metabolic activation of xenobiotics was further considered in Section 2. Much evidence was (rightly) placed on the role of cytochrome P450s, the topics discussed being the influence of the anticarcinogen dehydroepiandrosterone on P450, the pharmacogenetics and polymorphism of P450's, and the aromatase cytochrome P450 complex. The possible contribution of (non-cytochrome P450 dependent) oxygen radicals to bioactivation of carcinogens is a further important parameter, which was discussed using benzo(a)pyrene as an example. Other bioactivation processes that were reviewed were those involving glutathione conjugate formation (e.g. 1,2-dihaloalkanes), acetyltransferase and sulphontransferase (aromatic amines). Surprisingly, epoxide hydrolase, which is involved in epoxide detoxification and which is polymorphically expressed, was not given much coverage, although the importance of the N-acetylation phenotype to the carcinogenic effect of arylamines was included.

Section 3, which partially overlaps with Section 2, is about gene regulation of xenobiotic metabolising enzymes. In a field moving as fast as this there are naturally some areas which have progressed since the reviews were written and one can find omissions such as $\gamma$-glutamyl transpeptidase. However other areas which are well reported are the regulation of cytochrome P-450, the elegant studies on the nuclear glucocorticoid receptor, regulation of DT-diaphorase, and expression of glutathione transferases. The important effects of xenobiotics on signal transduction and $Ca^{2+}$ concentrations, (although not particularly appropriate to the section title), are also discussed.

Resistance processes to cancer drugs from the basis of Section 4. Resistance to many drugs is associated with the expression of a 170 kD glycoprotein (P-glycoprotein), whose function is clearly reviewed. Exciting prospects exist for anticancer agents which could block the transport activity of P-glycoprotein. Other enzymes involved in resistance which are discussed are glutathione transferases (GST), glyoxalase I and UDP-glucuronosyl transferases. The first of these enzymes appears under different roles in each of Sections 2, 3 and 4. Although it might have been a challenging task for a contributor, an overall summary with a unifying hypothesis on function, polymorphism etc. of GST might have been interesting to include.

The next shorter section covers xenobiotics as a cause of human cancer. Three papers cannot even scratch the surface of this subject, and indeed only one class of compounds (the hetrocyclic amines) are considered in detail. (It however maybe appropriate to choose these as the sole example in this volume, in view of the pioneering work carried out by the Japanese on hetrocyclic amines in cooked food.) To make up for the deficiency in covering other compounds there is a penetrating and though-provoking review by Ames *et al.* pointing out the importance of concentrating our attention on natural chemicals, in addition to the synthetic chemicals which have previously occupied many regulators' minds.

One real possibility for determining which xenobiotics are involved in human cancer initiation rests in the novel disciplines of 'biomonitoring', reviewed in Section 6. These techniques allow the detection of carcinogen adducts with DNA at levels down to ca. 1 modified DNA base per cell. Screening systems to monitor qualitatively and quantitatively exposures to mixtures of carcinogens are being developed using measurements of both DNA and protein (especially haemoglobin) adducts. Four papers review the major techniques employed ($^{32}P$-postlabelling, immunoassays, gas chromatography/mass spectrometry) for DNA and protein adduct measurements, and the relation of these data with mutations and chromosomal changes is discussed. This is a most interesting section of the book, and an appropriate one for its conclusion. Again because of the pace of progress in this area we know now of interesting novel findings, for example relating to mutation of the p53 gene, which could not have been included at the time of writing. This sadly is unavoidable in a publication now of a meeting held 18 months ago.

The book is notable for its high quality of editing and would certainly be a valuable addition to the bookshelves of chemical or biological carcinogenicists. The range of topics reviewed, although not comprehensive, is wide and the book is a useful source of up to date knowledge on molecular findings in chemical carcinogenesis.

P. Farmer